# Cancer-related multiple brain infarctions caused by Trousseau syndrome in a patient with metastatic colon cancer: a case report

**DOI:** 10.1186/s40792-016-0217-7

**Published:** 2016-09-05

**Authors:** Takahiko Akiyama, Yuji Miyamoto, Yasuo Sakamoto, Ryuma Tokunaga, Keisuke Kosumi, Hironobu Shigaki, Junji Kurashige, Masaaki Iwatsuki, Yoshifumi Baba, Naoya Yoshida, Hideo Baba

**Affiliations:** Department of Gastroenterological Surgery, Graduate School of Medical Sciences, Kumamoto University, 1-1-1 Honjo, Kumamoto, 860-8556 Japan

**Keywords:** Metastatic colorectal cancer, Brain infarction, Trousseau syndrome, Hypercoagulability

## Abstract

Thromboembolism that occurs in association with a malignant tumor is known as Trousseau syndrome. We herein present a case of Trousseau syndrome during systemic chemotherapy for metastatic colon cancer. A 65-year-old man with multiple liver metastases underwent primary tumor resection and systemic chemotherapy. Multiple brain infarctions were detected by magnetic resonance imaging immediately after first-line chemotherapy, which was deemed ineffective. There was no evidence of cardioembolic stroke or carotid atherosclerosis. Although the patient was initially asymptomatic, he subsequently developed paralysis. Despite anticoagulant treatment, he developed repeated recurrences of the infarction, and the area of the infarction spread as the liver metastases progressed. The patient’s condition showed no response to an alternative treatment regimen for advanced colon carcinoma. He died approximately 11 months after tumor discovery.

## Background

Malignancy can produce a hypercoagulable state associated with a significant risk of thrombosis. Trousseau syndrome, which was first reported in 1865 [1], is defined as unexplained thrombotic events that precede the diagnosis of an occult visceral malignancy or appear concomitantly with the tumor [2]. The pathogenesis of Trousseau syndrome involves the interplay of multiple variables: age, bed rest, infection, surgery, drugs, and procoagulants secreted by tumor cells or normal host tissue. We herein report a fatal case of Trousseau syndrome caused by metastatic colon cancer.

## Case presentation

**Fig. 1:**
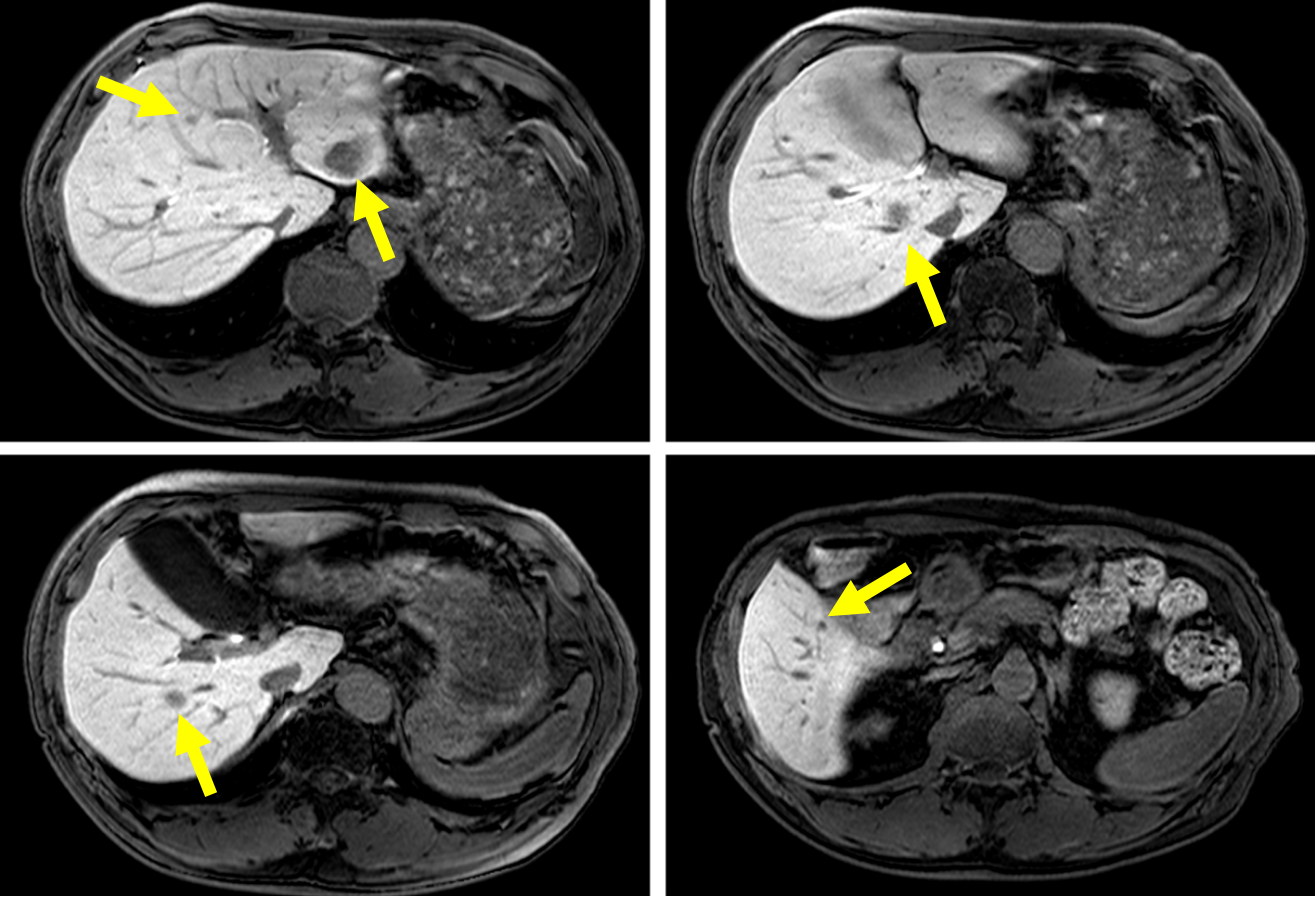
Gadoxetic acid (Gd-EOB-DTPA)-enhanced MRI for detection of liver metastases showed multiple liver metastases before chemotherapy

**Fig. 2:**
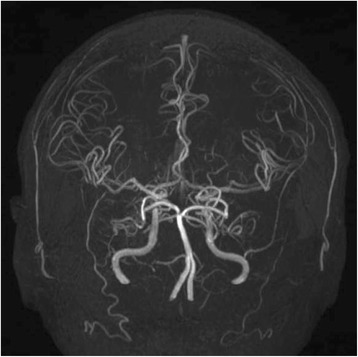
Brain and carotid artery magnetic resonance angiography showed that the main vessels were normal

**Fig. 3:**
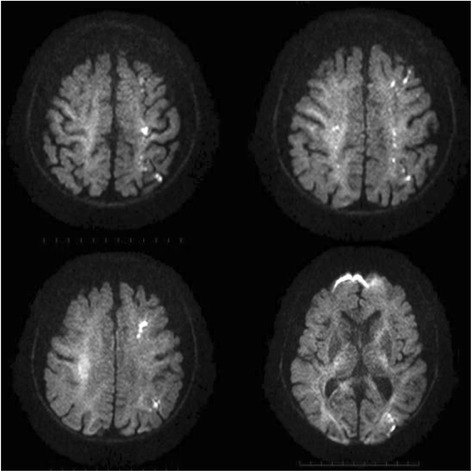
Brain MRI (diffusion-weighted) shows multiple small brain infarctions

**Fig. 4:**
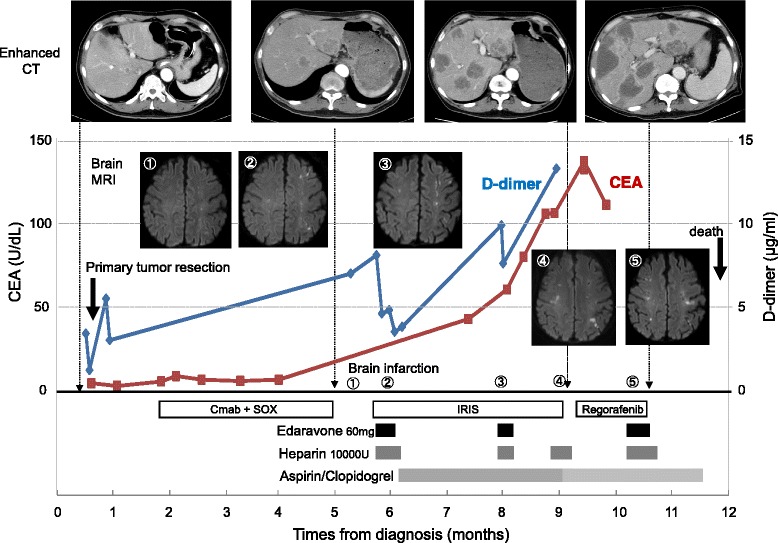
Clinical course of the patient’s laboratory data, enhanced CT for detection of liver metastases, brain MRI for detection of infarctions, anticoagulant therapies, and anticancer therapies. *Cmab + SOX* cetuximab, oxaliplatin, and S-1 therapy, *IRIS* irinotecan and S-1

A 65-year-old man presented to our emergency department with clinical dehydration and disturbance of consciousness. Systemic computed tomography showed obstructive ileus secondary to an ascending colon tumor, and he was diagnosed with ascending colon cancer with multiple liver metastases (Fig. 1). Insertion of an ileus tube and administration of intravenous therapy improved his hydration and level of consciousness. After the patient’s condition improved, we performed a right hemicolectomy. The postoperative pathological examination revealed penetration of cancer cells into the subserosa, subserosal vessels, and subserosal lymphatic ducts. Histological examination revealed well-differentiated tubular adenocarcinoma. Metastasis was detected only in the paracolic lymph nodes. After primary tumor resection, he received systemic chemotherapy comprising oral fluoropyrimidine (S-1), oxaliplatin, and cetuximab in a clinical trial setting. However, his disease became progressive within 3 months; therefore, we planned to perform second-line chemotherapy (irinotecan + S-1). Before he started the second-line chemotherapy, he was diagnosed with a silent brain infarction based on brain magnetic resonance imaging (MRI) findings following the clinical trial criteria. Although he exhibited no associated symptoms, brain MRI showed multiple bilateral small cerebral infarcts and essentially normal MR angiography (Fig. 2). He was carefully examined for evidence of cardioembolic stroke or carotid atherosclerosis. However, Holter electrocardiography, echocardiography, and carotid ultrasonography revealed no abnormal results. Therefore, the infarction was considered to be most likely caused by its relationship to cancer (Trousseau syndrome). Because of the risk of hemorrhage and lack of symptoms, the infarction was not treated with anticoagulant drugs first. Although the patient’s progress had been stable, paralysis of the right upper limb occurred 1 week later. MRI showed that the area of the infarction in the left cerebral hemisphere had spread (Fig. 3). Both the fibrin degradation product and D-dimer levels continued to increase, and treatment with edaravone and aspirin was begun. Although the anticoagulant therapy was continued to prevent recurrence, the patient experienced repeated recurrences of the infarction with limb paralysis during the second-line chemotherapy. MRI showed that the infarction area in the cerebral hemisphere had spread with the progression of liver metastases (Fig. 3). In addition, both the serum fibrin degradation product and D-dimer levels had continuously increased along with an increasing serum carcinoembryonic antigen level (Fig. 4). Despite the possibility of thrombosis, we carefully administered regorafenib as third-line chemotherapy because of its anticancer effect. However, there was no response to the regorafenib. The patient therefore received best supportive treatment and died 1 month later.

### Discussion

We have presented a case involving a patient with Trousseau syndrome during systemic chemotherapy for metastatic colon cancer. The patient developed repeated recurrences of the infarction with the progression of liver metastases.

Trousseau syndrome is defined as unexplained thrombotic events that precede the diagnosis of an occult visceral malignancy or appear concomitantly with the tumor [2]. However, the term Trousseau syndrome is often applied to patients with malignancy who develop some form of thrombosis because of a thrombotic tendency such as immobility, dehydration, mechanical compression of veins, an infectious processes, or chemotherapy.

Although the molecular mechanism of Trousseau syndrome is not completely understood, it involves the interplay of multiple variables. Early reports suggest that tumors associated with thrombotic complications were mucin-secreting adenocarcinomas of the gastrointestinal tract [3]. However, not all cases of Trousseau syndrome are associated with mucin-producing carcinomas. A number of substances may contribute to the thrombotic tendency in patients with cancer. The main contributors that have been isolated from human tumors with procoagulant activity are classified into two major categories: tissue factor (also called thromboplastin) and cancer procoagulant [2, 4]. Tissue factor forms a complex with factor VIIa to activate factors IX and X, thereby initiating the coagulation protease cascades. Tissue factor is a cell surface receptor for factor VII and is found more frequently in epithelial malignant tumors; pancreatic tumors tend to secrete particularly high levels of tissue factor.

In one study of the infarct pattern in patients with cancer who developed stroke, diffusion-weighted imaging patterns of multiple lesions involving multiple arterial territories were more frequently observed in patients with cancer, whereas single/multiple lesions involving one arterial territory were observed more frequently in patients with conventional stroke mechanisms such as atherosclerosis, cardioembolism, or lacunar stroke [5].

In the present case, both first- and second-line chemotherapy were ineffective; even with third-line chemotherapy, the patient’s overall survival was only 11 months, which is shorter than the current median survival in patients with metastatic colorectal cancer. The association between coagulation abnormalities and a poor prognosis of colorectal cancer has been reported [6]. Once brain infarction has occurred in a patient with cancer, the overall prognosis is poor. The median survival is only 4.5 months, and survival is strongly correlated with initial neurologic disability, similar to the general population with stroke. Furthermore, several reports have evaluated the efficacy of chemotherapy or radiotherapy and the presence of pretreatment coagulation abnormalities in cancer therapy. Such evidence would help oncologists and their patients to make treatment decisions.

The standard approach to Trousseau syndrome is early initiation of an anticoagulant such as a heparin-containing product. The decision to anticoagulate a patient with a limited life expectancy can be difficult because of the need for close laboratory monitoring and the risk of bleeding. The most common therapy for patients with brain infarction is prevention of dehydration and administration of anticoagulation therapy by warfarin in the acute phase and warfarin in the chronic phase. However, some oral fluoropyrimidines, which are key anticancer drugs for treatment of colorectal cancer, potentiate the effect of coumarin derivatives, inducing hemorrhagic adverse events. In several reports, low-molecular-weight heparin was successfully substituted for unfractionated heparin in the management of Trousseau syndrome, in part because of its improved pharmacokinetics, the ability to give single daily doses, and the reduced incidence of heparin-induced thrombocytopenia [2, 7]. In addition, it should be remembered that anticancer therapy for the causative tumor, if able to be performed, is equally important. We also used antiplatelet agents, aspirin, or clopidogrel. Antiplatelet therapy is used for both the management of acute ischemic stroke and for the prevention of stroke [8]; however, the effect of antiplatelet therapy for Trousseau syndrome is unknown. Some oral factor Xa inhibitors have recently become widely used in the clinical setting. The combination of anticancer therapy and oral Xa inhibitors or antiplatelet therapy is recommended for treatment of patients with Trousseau syndrome.

## Conclusions

During daily clinical practice, the possibility of Trousseau syndrome should be considered in patients with sudden stroke, and a better understanding of cancer-associated hypercoagulation is needed.
